# Optimisation of perioperative procedural factors to reduce the risk of surgical site infection in patients undergoing surgery: a systematic review

**DOI:** 10.1007/s44250-023-00019-9

**Published:** 2023-02-13

**Authors:** P. Calò, F. Catena, D. Corsaro, L. Costantini, F. Falez, B. Moretti, V. Parrinello, E. Romanini, A. Spinarelli, G. Vaccaro, F. Venneri

**Affiliations:** 1 University Teaching Hospital of Cagliari and Surgical Department at University of Cagliari, Cagliari, Italy; 2 Department General and Emergency Surgery at Bufalini Hospital, Cesena, Italy; 3 International Research at BHAVE, Via GiambattistaVico 1, 00196 Rome, Italy; 4 Department of Medical and Surgical Sciences, School of Community Medicine and Primary Care, University of Modena and Reggio Emilia, Modena, Italy; 5 Department of Orthopaedics ASL Roma 1 and Director UOC Orthopaedics Hospital San Filippo Neri, Rome, Italy; 6 Orthopedics and Traumatology Complex Operative Unit, University Teaching Hospital of Bari Polyclinic, Bari, Italy; 7 Operative Unit of Quality and Clinical Risk Manager at “G.Rodolico-San Marco” University Teaching Hospital in Catania, Catania, Italy; 8 SIOT Guidelines Commission, Rome, Italy; 9 Complex Operative Unit of Orthopedics and Traumatology at University Teaching Hospital of Bari Polyclinic, Bari, Italy; 10 Operative Unit of Orthopedics and Traumatology at University Teaching Hospital of Bari Polyclinic, Bari, Italy; 11 Social, Epidemiological and Outcome Research at BHAVE, Via Giambattista Vico 1, 00196 Rome, Italy; 12 Sociologist UO Education and Health Promotion, Asp Catania, Via Santa Maria la Grande 5, 95124 Catania, Italy; 13 Simple Structure Clinical Risk and Surgical Emergency in Florence, Florence, Italy

## Abstract

Surgical site infections (SSI) are the leading cause of hospital readmission after surgical procedures with significant impact on post-operative morbidity and mortality. Modifiable risk factors for SSI include procedural aspects, which include the possibility of instrument contamination, the duration of the operation, the number of people present and the traffic in the room and the ventilation system of the operating theatre.The aim of this systematic review was to provide literature evidence on the relationship between features of surgical procedure sets and the frequency of SSI in patients undergoing surgical treatment, and to analyse how time frames of perioperative processes and operating theatre traffic vary in relation to the features of the procedure sets use, in order tooptimise infection control in OT. The results of the systematic review brought to light observational studies that can be divided into two categories: evidence of purely clinical significance and evidence of mainly organisational, managerial and financial significance. These two systems are largely interconnected, and reciprocally influence each other. The decision to use disposable devices and instruments has been accompanied by a lower incidence in surgical site infections and surgical revisions for remediation. A concomitant reduction in post-operative functional recovery time has also been observed. Also, the rationalisation of traditional surgical sets has also been observed in conjunction with outcomes of clinical significance.

## Introduction

Surgical site infections (SSI) are the leading cause of hospital readmission after surgical procedures [1] with significant impact on post-operative morbidity and mortality [2] Modifiable risk factors for SSI include procedural aspects, which include the possibility of instrument contamination, the duration of the operation, the number of people present and the traffic in the room [3], and the ventilation system of the operating theatre [4].

These factors influence each other because preparation of instrumentation involves processes that involve a certain number of operators, each step increases the risk in contamination, and the need for different instrumentation, depending on the type of operation, can lead to altered traffic in theatre.

The need to optimise infection control in OT is accentuated by the SARS-CoV-2 pandemic and the need to prevent and respond to future epidemics.

### Research questions

1. What is the relationship between the features of surgical procedure sets and the frequency of surgical site infections (SSI) in patients undergoing surgical treatment?
2. How do the time frames of perioperative processes and operating theatre traffic vary in relation to the features of the procedure sets used?
3. What is the impact of streamlining and optimising surgical procedure sets and their direct and indirect costs?

## Methods

This systematic review is conducted in accordance with the *Preferred Reporting Items for Systematic Reviews and Meta-Analyses—PRISMA Statement 2020* [5].

Accordingly, the literature review process was carried out in the following stages:

1. Formulation of research questions by adapting the PICOS model
2. Developing a search string applied to databases;
3. Collection of identified records;
4. Screening of records according to inclusion criteria;
5. Full-text selection of the studies identified during screening;
6. Data extraction from the studies included;
7. Thematic analysis of results

### Research strategy

The search was applied to the following databases: MEDLINE, Embase, Web of Science, CINAHL, The Cochrane Library.

The search string was formed by combining the following terms:

1. Procedure or procedural or surgery or surgical.
2. Tray or pack.
3. 1 and 2

The complete search strategy can be found in Appendix 1.

### Selection of studies

An initial screening was performed on the title and abstracts of the Records identified in the databases. Subsequently, the selected records were analysed on full text for inclusion or exclusion. The selection was made according to the following criteria:

- **Participants**: surgical specialists, operating theatre (OT) nurses and nurse coordinators, theatre technicians, sterilisation centre (SC) coordinators and operators, other personnel with clinical, organisational, or logistical roles; patients undergoing surgical treatment
- **Intervention:** use of conventional or disposable procedure sets; processes of rationalising surgical sets
- **Outcome**: incidence of SSIs, procedure time, in/out traffic of personnel, costs related to surgical procedures
- **Setting**: general and specialist surgery facilities, supply services and sterilisation centres (SC)
- **Study designs**: qualitative, observational, nRCTs and RCTs, systematic reviews, guidelines

English-language studies published up to 28.12.2021 were selected.

Any previous guidelines, systematic reviews or meta-analyses discovered during the database search, concerning surgical sets from the point of view of clinical risk reduction, procedure optimisation and the rationalising of instruments, were selected and summarised in a separate table.

### Data extraction

The data are extracted from the studies in a specially designed worksheet. Data were extracted on the test population, the surgical setting, the procedures performed, the operations and any follow-ups. The main operational outcomes of interest were:

1. A reduction in the incidence of post-surgical SSIs;
2. A reduction in procedure times;
3. A reduction in operating theatre traffic flow;
4. A reduction in costs associated with the intervention.

## Results

**Fig.1:**
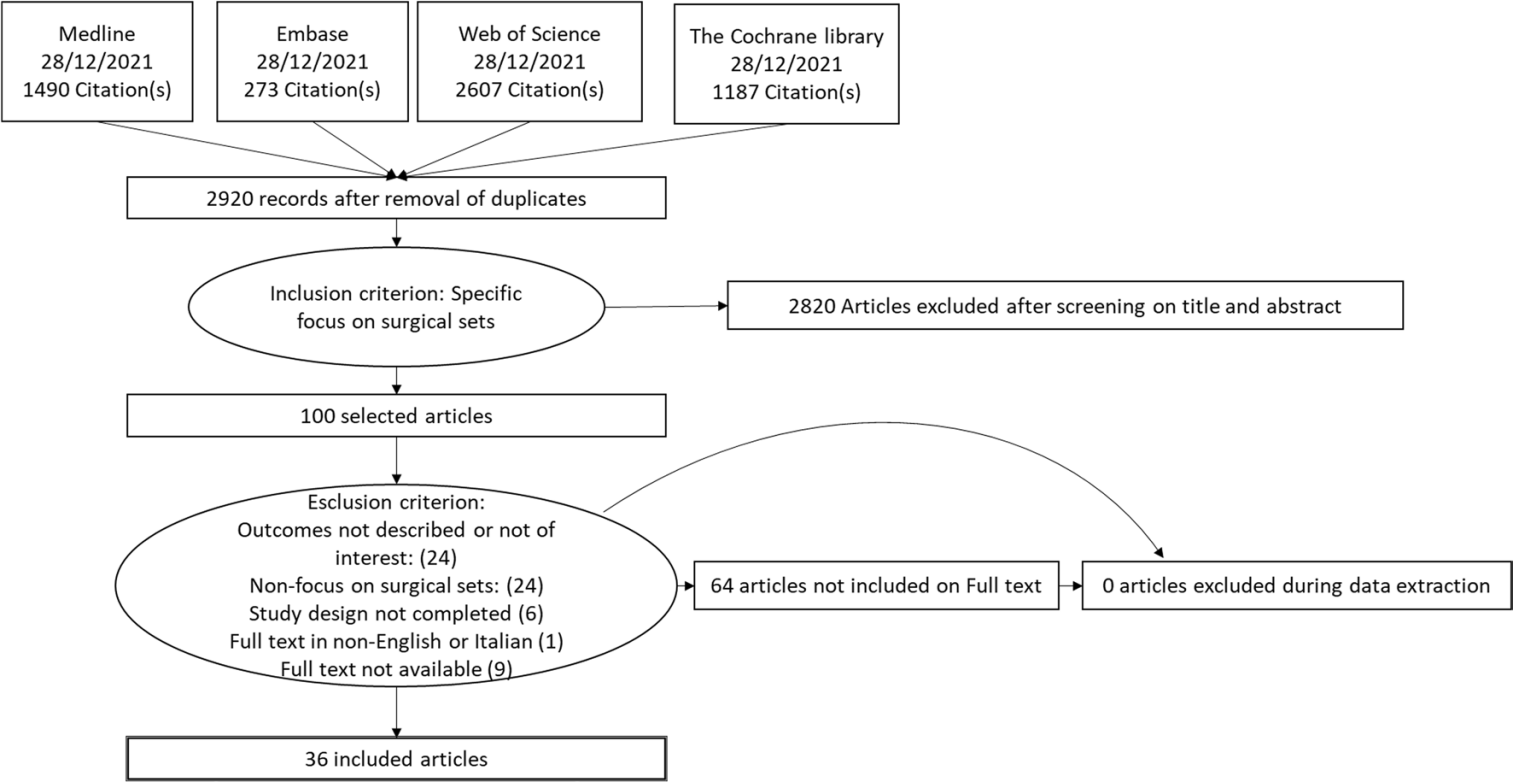
PRISMA flow diagram

Figure 1 represents the flow of the literature review effected. A search through PubMed, Embase, CINAHL, Web of Science and The Cochrane Library identified 5 865 records. Five further studies were added manually after consulting the bibliographies of the articles analysed. After eliminating duplicates, the remaining records were screened by title/abstract. 98 records were assessed as eligible and analysed on full text.

A total of 62 studies were excluded from the complete analysis. In conclusion, 36 studies were eligible for inclusion in the review.

A complete list of selected studies and the relevant bibliography can be found in Appendix 2.

Excluded, reasons for categorisation: > 62:

- full text not available (9): Littell 1951, Sebben 1988, Sheth 2003, Dieryck 1998, Stephanie 2010, Akridge 2004, Osborne 1999, Passey 2002, Wilkie 1986
- Outcome not described or not of interest: (24) Ahmadi, 2019; Egan 2021, Baskett 2004, Bhumisorikul 2004, Costa 2018, Fogliatto 2018, Glaser 2015, Halbert 1988, Huang 2021, Kimse 2021, Kusuda 2016, Strulak 2021; Strzelecki, 1989, Torres 2021, Bradley 2019, Alfred 2021, Dana Barlow 2015, Holdsworth 2021, Igesund 2019, Navi 2012, Parker 2006, Wells 2017, Wells 2018, Eggleston 1997
- No focus on surgical sets: (24) Agarwal 2019, Arslan 2018, Dilworth 1992, Edilich 1992, Fadaak 2021, Haya 2018, Johnson 2016, Kaygusuz 2003, Khurana 2018, Kong 1994; Kwaan 2016; Lin 2018, Martinez 2020; McDermott 2016; Meals 2007; Moccia 2020; Nguyen 2019; Panahi 2012; Rao 1992, Wagner 2021, Weiser 2018; Watters, 2011; Makram 2021, Olivere 2021
- Study design not covered (e.g. editorials, case reports, opinion papers) (6): Reams 2013, Weber 1998, Nadeau 2018, O'Donnell 2002, Stephanie 2010, Goldberg 2019
- Full text in other language (1): Blanc 2017

Including: 36:

- Previous reviews on the subject (4)
- Primary studies (32)

### Summary of evidence prior to current review

Four literature reviews relevant to the scope of the current work were identified (Table 1). In particular, Dos Santos and colleagues [6] sought to understand the main techniques and approaches in the rationalising of surgical sets, the impact on financial and operational performance, and the knowledge gaps towards which research should be directed. The review identified many studies reporting signs of improved performance both *operationally*, concerning set assembly, OR management and ergonomics, and *efficiently* in terms of washing and sterilisation processes, repair, purchasing, set-up and professional education. The authors mapped an outline for future research. Salient points to be explored are: the promotion of consensus building in multidisciplinary teams, participation in surgical set rationalisation projects and consolidation of the progress achieved; technologies for instrument tracking; cross-surgical set analysis aimed at instrument reduction; relocation of instruments excluded from sets; how to measure “non-material” and “non-tangible” benefits of set rationalisation and set safety after the process; objectives following the establishment of a surgical set rationalisation cycle.

**Table 1:** Previous systematic reviews

Author, Year	Targets	Criteria	Studies included	Results
Bussiéeres et al. 2017 [8]	Examining the risks and benefits of opening surgical sets in the operating room	Impossible to evaluate	One experimental study, four clinical guidelines	The contamination rate in the first 30 min of opening sets was 4% in an experimental study The guidelines recommend that sets should be opened as close to their use as possible, without precise indications as to the time interval
Schomig et al. 2020 [7]	Assess preoperative and intra-operative causes of contamination of instruments and implants, and possible solutions	Experimental and observational studies, in English, conducted in humans, focusing on the contamination of instruments and implants in orthopaedic and spinal surgery Excluding case reports, opinion papers, grey literature	Nine studies on preoperative causes, 26 on intra-operative causes	Contamination rates of instruments still significant; possible ineffectiveness of sterilisation alone; importance of changing gloves before placing implantable devices; disposable kits as an opportunity to reduce contamination; questionable applicability in the clinical context of recommendations concerning reprocessing of devices
Dos Santos et al. 2021 [6]	Review existing literature on surgical tray rationalisation (STR) Summarising techniques and approaches for STR Examine the impact of the STR in operational and cost aspects	Inclusion: English language; observational or experimental; quantitative, qualitative or mixed methodologies Exclusion: studies not in conformity with the questions; studies concerning instrument cleaning, contamination reduction, instrument cleaning guidelines, sector layouts, professional training and types of washing	48 articles consisting of expert analysis (n = 34), *lean practice* protocol (n = 9), mathematical programming (n = 5) 14 surgical specialities; 116 procedures	Operational improvements concerning the set assembly process (n = 16), operating theatres (n = 26), ergonomic functionality (n = 16) Cost savings in sterilisation (n = 36), instrument repair (n = 15), purchasing (n = 22) Development of a flowchart for the rationalisation process for surgical sets Drafting nine questions to feed into future research
Dekonenko et al. 2020 [9]	Standardisation and cost-effectiveness of paediatric surgery sets	English language Outcome of reducing surgical sets Primary studies Human studies	Five prospective observational studies Paediatric surgical sub-specialities: general surgery, thoracic surgery, urology, orthopaedics, neurosurgery,	Stakeholder involvement: multidisciplinary consensus meetings (n = 3), development of standardised preference cards (n = 3), real-time feedback on the costs of choices made (n = 1), observer researchers in OT (n = 1), pre- and post-optimisation feedback questionnaires (n = 3) Measures of savings: different methods (n = 3) heterogeneously classified processing cost (n = 2) Clinical safety: no significant changes in duration of surgery, length of hospital stay, frequency of complications, use of postoperative imaging (n = 3) Stakeholder satisfaction: nurses, surgeons, technicians(n = 1)

A systematic review [7] analysed the problem of contamination of implantable instruments and devices in spinal surgery. Many studies have shown significant contamination rates. Preoperative factors, mainly related to sterilisation and device handling processes, and intra-operative factors, more dependent on personnel procedures, were noted. With respect to pre-operative factors, several studies question the overall effectiveness of sterilisation alone. At the same time, recommendations for device reprocessing are recognised as being difficult to implement in clinical settings. Regarding intra-operative processes, studies emphasise the importance of preventive practices such as changing gloves before handling implant materials, because the risk of contamination increases with exposure time [7].

### Clinical interventions to prevent surgical site infections

Three observational studies report direct evidence of an effect on the incidence of SSI (Table 2). A cohort study conducted on 233 women with ovarian and uterine neoplasms who underwent colon surgery showed a statistically significant reduction in SSIs 30 days after surgery after implementation of a preventive bundle targeting the surgical wound closure phase, including the use of a separate surgical set [10]. Two other observational studies investigated the use of disposable sets versus traditional reusable sets. The first, conducted on neuro-surgical patients undergoing lumbar fusion, observed a reduction in the incidence of SSIs from 6% in the series of 100 patients treated with reusable instruments to 2% in the series of 49 patients treated with disposable sets (p < 0.001). A significant reduction in surgical procedure duration (p < 0.05) and functional recovery time (p < 0.001) was also observed [11]. In the second, 449 orthopaedic patients underwent total knee arthroplasty using single-use instrumentation and only 0.2% underwent revision surgery for SSIs, compared to 3% (p < 0.006) of cases in a series of 169 patients operated on with conventional instrumentation [12].

**Table 2:** Clinical interventions to prevent surgical site infections

Author, Year	Demographic characteristics/interactors	Intervention	Study design	Sample Size	Results
Schiavone et al. 2017 [10]	Women between 22 and 87 years of age with predominantly ovarian (77–78%) and uterine (19–15%) tumours undergoing colon surgery; ASA score 3 or 4 in most cases	Multi-intervention bundle of peri-operative preventive procedures including use of separate surgical sets for surgical wound closure, disinfection with chlorhexidine gluconate and glove change before closure	Prospective cohort	n = 233 PRE cohort: n = 115 POST cohort: n = 118	Incidence of SSI at 30 days PRE: 43/115 (37%) POST: 14/118 (12%), p ≤ 0.001
Litrico et al. 2016 [11]	M = 21; F = 28 Average age: 61.6/ ± 12.8, (29–84) Sufferers of degenerative inter-vertebral disc disease, spinal canal stenosis, degenerative spondylolisthesis. Neurosurgery	Use of disposable (intervention) or reusable instrumentation in standard packaging (control) for performing posterior lumbar fusion (PLIF) or transforaminal fusion (TLIF)	Observational cohort, bi-centric	Disposable: N = 49 Reusable: n = 100	Frequency of SSI: - Disposable: 1/49 (2%) [individual risk factors: diabetes mellitus 2, obesity] - Reusable: 6/100 (6%) [individual risk factors: obesity (2/6, 33%), diabetes (1/6, 16%) duration of the procedure: -15 min (p < .05) Difference in functional recovery between the two series (Oswestry Disability Index, ODI): + 21.8 ± 14.1, p < .001
Siegel et al. 2015 [12]	Patients undergoing total knee arthroplasty surgery	Performing total knee arthroplasty using disposable instrumentation	Retrospective cohort	Traditional: n = 169 Single-use: n = 449	Frequency of surgical revision for SSI: 0.2% vs. 3% (p.006) OT set-up time: − 15’ (P < .05) cleaning time in OT: − 14’ (P < .05) duration of procedure: unchanged

### Procedures for surgical tray rationalisation

*Twenty-nine surgical tray rationalisations (STR)* as part of quality improvement projects aimed at creating an optimised set for specific procedures.

In this context, unused items were removed from the instrument management process.

The studies presented in the literature (Table 3) involved multiple branches, including: orthopaedics (n = 6), gynaecology (n = 4), ENT (n = 6), thoracic surgery, endocrine surgery, paediatric surgery (n = 2), neurosurgery, ophthalmology, vascular surgery, breast surgery (n = 2), urology, general surgery (n = 2), hand surgery, and plastic surgery. Several studies included an observation phase involving the preparation, use and reprocessing of the surgical sets between the operating theatre (OT) and the sterilisation centre (SC) (n = 13).

**Table 3:** Procedures for rationalising traditional surgical trays

Author, year	Participants and Context	Process	Scope of the intervention
Byrnes et al. 2017 [18]	Pelvic and re-constructive gynaecological surgery specialists OT Staff	Creation of a set for minor gynaecological surgery adapted from the standard vaginal hysterectomy set: 1) Elimination of unused tools on a subjective basis; 2) Formal audit of the use of tools	Instruments in the vaginal hysterectomy set: n = 67 Estimated processing cost: $0.51/instrument
Capra et al. 2019 [19]	Patients undergoing total hip (51%) or knee (49) arthroplasty	Design and introduction of surgical sets for total hip or knee arthroplasty	n = 96 Pre-introduction: 38 post-introduction: 58
Crosby et al. 2020 [20]	N.A	Optimisation of surgical sets for ENT surgeries	Set-up: 96 sets Assembly: 56 sets
Cichos et al. 2017 [15]		Optimisation of surgical sets for mediastinoscopy, VATS, robotic thoracic surgery and thoracotomy by three thoracic surgeons of a university department	Estimates made considering the number of operations carried out in the department in 2016: 398 VATS; 231 robotic procedures; 163 thoracotomies; 162 mediastinoscopies
Cichos et al. 2019 [21]	N.A	Optimisation of 23 surgical sets for total arthroplasties by 15 surgeons and 1 health professional	N.A
Chin et al. 2014 [22]	ENT surgeons	Observation of instrument use in 5 common ENT procedures Creation of an optimised tray including only the tools used at least 20% of the time Review of the tray by specialists	Observed sets: n = 226
Dobson et al. 2015 [23]	N.A	Discussion with three professionals to identify preferences in the composition of individual kits; development of a heuristic mathematical model predictive of savings; Testing the model against data from operating diaries	500 cases of surgical kit set-up preferences
Dyas et al. 2018 [16]	N.A	Creation of a surgical set for thyroidectomy and parathyroidectomy on the basis of a consensus of a multidisciplinary team by rationalising the head and neck surgery set; Evaluation of the new set by two endocrine surgery specialists; Estimated savings in comparison with use of the head-neck set	13 sets of head and neck surgery Historical data: 900 thyroidectomy/parathyroidectomy operations per year
Farrelly et al. 2017 [24]	5 paediatric surgeons Central sterile processing staff OT nurses Surgical technicians Material management staff	Interdisciplinary review of the individual instruments included in the 20 sets	21 surgical sets
Farrokhi et al. 2013 [25]	Minimally invasive spinal surgery (MIS) and deep brain stimulation (DBS) procedures Average age: Minimally invasive spinal surgery: 58 (16–93) craniotomy/SCP: 61 (26–92) Surgeons, OT nurses, surgical technicians, nurse coordinators, sterilisation centre operators	Direct observation of the preparation and use of instruments between the sterilisation centre and operating theatre Optimisation through Lean methodology with a multidisciplinary approach: 1) sort: OT nurses track the use of instruments 2) simplify: elimination of unused instruments 3) sweep: review by all members 4) standardise: generation of a new optimised set 5) self-discipline: new usage tracking and regular reporting of usage data	MIS cases: n = 885 Open spinal surgery cases: n = 156 DBS cases n = 134
Fu et al. 2021 [26]	Five common ENT procedures (adenotonsillectomy, myringotomy, septoplasty, Endoscopic sinus surgery, thyroidectomy)	1) Involvement of stakeholders with training and problem-sharing sessions 2) fortnightly feedback loop	Six surgeons Ten months
Grodsky et al. 2020 [27]	Patients undergoing vitrectomy Specialist surgeon Operating theatre nursing staff Central Sterile Processing department	Rationalisation of vitrectomy surgical set	Observation in 169 patients
Harvey et al. 2017, [28]	Departments of Pelvic Medicine and Women's Re-constructive Surgery and of Minimally Invasive Surgery Peri-operative staff Specialist surgeons Otolaryngologist	Identification of the 5 most frequently used surgical sets by theatre staff; Categorisation of the extent of use of each instrument by all surgeons, using a group consensus model	367 instruments Period of analysis: 9 months Sets analysed: n = 39
John-Baptiste et al. 2016, [29]		Comparison of the cost of a redundant set and a rationalised set according to the study by Chin et al. 2014	
	Set for 5 common procedures		
Knowles et al. 2021, [30]	Vascular surgeon 6 surgeons	Lean method observation of cases in OT analysis with OpFlow software discussion of analyses with multidisciplinary team (surgeons, technical coordinators, sterilisation centre coordinators) implementation of optimised sets	Cases: N = 168
Koyle et al. 2015, [31]		Rationalisation of inguinal hernia repair sets by paediatric surgeons and urologists: Phase I—observation Phase II—standardisation and evaluation in OT and central warehouse	Professionals involved: 8 surgeons, 6 urologists Operations studied in phase I: 58 (12U + 44S) Operations studied in Phase II: 52
Lonner et al. 2021, [32]	Total hip (THA) and knee (TKA) arthroplasty operations performed by four surgeons	Observation of the processes of use and sterilisation of surgical sets in the operating theatre and in the sterilisation centre; discussion and rationalisation using the Lean method	35 observed interventions 1277 TKA /year 270 THA /year
Malone et al. 2018, [33]	Three breast cancer surgery specialists Quality improvement coordinator OT staff (nurses, technicians) Sterilisation centre	Creation of a rationalised surgical set for segmental resection of the breast from a generic head-and-neck surgical set	13 sets at the start 98 instruments Interventions/year n = 449
Marchand et al. 2020, [34]	Interdisciplinary staff (orthopaedic surgeons, technicians, OT and sterilisation centre coordinators)	Observation of set-up and cleaning in OT Observation of processes in sterilisation centre	Cohort (sterile containers): n = 185 Control cohort (wraps) = 44
Nast et al. 2019, [35]	urologists, surgical technicians, and representatives from the Central Processing Department	Plan, Do, Study, Act (PDSA) cycle for surgical cost reduction Observation of cases in a blind study and selection of instruments on the basis of utilisation rate (cut off: 20%) Interdisciplinary discussion of the rationalised list	10 inguinal orchiopexy with hernia cases, 10 scrotal orchiopexy cases, and 10 inguinal hernia cases Total of 54 cases observed
Schwartz et al. 2021, [36]	4 breast surgery surgeons	Cataloguing the 2 sets used in the main surgical procedures. Breast surgery questionnaire (surgeons' perception of use) and observation of 8 cases; development of 1 set rationalised according to the questionnaire and observation results	Pre cases: n = 239 Post cases: n = 210
Shim et al. 2021, [37]	Orthopaedic	Observation of cases Registration of instrument use Cost Analysis	37 cases 2 sets commonly used
Stockert and Langerman, 2014, [14]	Independent observer Different branches of surgery	Direct observation by a qualified researcher to quantify set utilisation in OT and the impact of unused instruments on manpower time spent in OT and sterilisation centre, and costs associated with sterilisation of instruments	49 procedures 237 sets 61 instruments in sterilisation centre
Thiel et al. 2019, [17]	Hand surgery	Creation of a rationalised set for hand surgery without sedation (wide awake); the specialist arbitrarily chooses whether to use the 'minimal' set or the standard [different technique]	Standard: n = 80 (45%) Minimal: n = 98 (55%)
Toor, J. et al. 2022, [13]	Orthopaedics, ENT, General surgery, gynaecology	Optimisation of tools using Kotter's change model to increase efficiency and reduce costs 1) Data Collection 2) Optimisation on a mathematical model 3) review of clinicians 4) application	
Tseng et al. 2020, [38]	Multidisciplinary team (surgeons, administrative staff, OT coordinators) 7 general surgery surgeons	Evaluation of supply costs for laparoscopic appendectomy operations performed Debriefing with the surgeon on each case carried out; information on costs of each instrument/supply and alternatives	Pre-intervention: n = 207 Post-intervention: n = 158
Van Meter et al. 2016, [39]	Gynaecological surgery	Observation of the use of instruments in the surgical context	Cases: N = 28 Set: n = 150 Specialists: n = 15
Wannemuehler et al. 2015, [40]	Adenotonsillectomy procedures	Lean method (LSS) 1) Questionnaire on surgeons' perception of instrument use 2) creation	Procedures: N = 700 Processed sets: n = 850
Wood et al. 2021, [41]	Plastic surgeon 17 surgeons OT and sterilisation centre staff Hospital management Surgical technicians Service line coordinators	Revision of 40 sets via a technological service (OpFlow) Intra-operative utilisation rate measurement Analysis of concordance of use between specialists by set and by procedure Development of an optimised set, reviewed by surgeons Implementation and change management process	Cases: N = 183 Two sets: generic, breast reconstruction Number of instruments per set: 109; 71

### Processes

The rationalisation process was preceded by training and sessions to raise awareness (n = 3) aimed at increasing the degree of motivation for change and overcoming resistance that was potentially risky for the entire process.

The extent to which an instrument is used, and therefore the benefits of keeping it in the set or not, was assessed on an objective basis by analysing the data collected during the observation (n = 13), with the cut-off of use generally considered to be 20% to 25%. In other cases, the selection was made on a subjective basis by professionals (n = 3), by consensus or the collection of questionnaires on perceived use. In one case a heuristic mathematical model was developed based on a discussion with skilled surgeons on their preferences as to the composition of individual sets [10]. The model was then tested on the operating division agendas with the goal of cost-cutting.

In some cases, only surgeons were involved (n = 6). In other cases, the formation of a multidisciplinary team was promoted (n = 7).

In most of the studies, the new optimised set was presented to the clinicians for review. Devices or instruments were then added based on their opinion.

Surgical devices or instruments that were excluded were generally packaged in a dedicated set, or the original set remained available and the frequency of use of the instruments excluded was used as a marker of the safety of rationalisation.

Some studies considered a follow-up aimed at assessing the degree of satisfaction with the new rationalised set after a period of time (n = 3).

### Outcomes

The outcomes analysed were mainly of three types:

- reducing the size of the set and the number of instruments (Table 4);
- reduction of peri-operative and sterilisation times and reprocessing of sets (Table 5);
- reduction of direct and indirect costs related to the procurement, replacement and reprocessing of instrumentation (Table 6).

**Table 4:** Outcome of rationalisation of surgical sets relative to instrumentation

Author, year	Indicators relating to instruments
Byrnes et al. 2017, [18]	Number of instruments: -19 (− 28%)
	Rate of instrument use in the vaginal hysterectomy set: 66%
Chin et al. 2014 [22]	Average instrument utilisation rate (I used/I total × 100): 27.8% (± 13.1)
	Optimised tray size: − 57%
Dobson et al. 2015 [23]	482 (96.4%) cases were solved using the heuristic model
Dyas et al. 2018 [16]	Number of new sets created: 3/13
	Number of instruments: 36 (-63%);
Farrelly et al. 2017 [24]	− 9 complete sets
	− 34% instruments per set
	− 1 826(-39.5%) instruments processed through the Central Sterile Processing (CSP) unit
Farrokhi et al. 2013 [25]	Reduction in instruments:
	MIS set: -139 (− 70%)
	DBS set: − 104 to − 72 (78–80%)
Fu et al. 2021 [26]	Utilisation rate of baseline instruments: 35%
	Average reduction in the number of instruments: 26%
Grodsky et al. 2020 [27]	− 89% Instruments
	Number of reopening episodes with the old set = 0
Harvey et al. 2017 [28]	− 70 (− 19%) instruments
	number of reopening episodes with the old set = 0
Knowles et al. 2021 [30]	Utilisation rate per set: 22.9% (vascular set), 12.5% (aortic set)
	Instrument reduction: − 45.8% (vascular set), − 62.5% (aortic set)
	Re-opening rate old tray: 0%
Koyle et al. 2015 [31]	Number of instruments not used in Phase I: 69/96 (U); 17/51 in (S)
	Frequency of use of the non-standardised set in Phase II: 3/52 (6%)
Lonner et al. 2021 [32]	Only 45.5% instruments used;
	Reduction in instruments after rationalisation: − 32.2%
Malone et al. 2018 [33]	51 instruments (− 48%)
	Frequency of reopening with the old set: 0%
Nast et al. 2019 [35]	pre-post utilisation rate:
	scrotal orchidopexy: 21.1% to 48.2%
	inguinal orchidopexy with hernia: 41.9% to 71.7%
	inguinal hernia: 32% to 62.3%
	reopening rate old tray: 10%
Schwartz et al. 2021 [36]	Utilisation rate: 38% (perceived) vs 55% (observed)
	Number of instruments: − 49%
	Set weight: − 33%
Shim et al. 2021 [37]	First tray: 10.6% unused instruments
	Second tray: 19.6% unused instruments
Stockert and Langerman, 2014 [14]	Average instrument (± SD) use rates were 13.0% for Otolaryngology (± 4.2%), 15.5% for Plastic Surgery (± 2.9%), 18.2% for Bariatric Surgery (± 5.0%), and 21.9% for Neurosurgery (± 1.7%)
Toor et al. 2022 [13]	Instrumentation measures:
	Total reduction of instrumentation: 35%
	Average reduction in set size: 18%
	Procedure cancellation rate: 3.9% (pre) to 0.2% (post)
	Satisfaction with surgical instruments (Likert scale 1–4): 1.7% to 80%
Van Meter et al. 2016 [39]	Average number of sets: 5.4 per individual case
	Average number of instruments per set: 38 (1–141)
	Utilisation rate: 20.5% (±2.8%)
	Significant difference between abdominal and vaginal cases
	No difference between laparoscopic or other approaches
Wannemuehler et al. 2015 [40]	Instrument reduction: − 54%
Wood et al. 2021 [41]	Number of instruments per set: − 40 (breast reconstruction), − 32 (generic)
	Instrument utilisation rate per set: 15.8% (generic set); 23.5% (breast reconstruction set)
	Rate of reuse of instruments removed after optimisation: 0% (generic set), 0.9% (breast reconstruction set)

**Table 5:** Outcomes of rationalisation of surgical sets related to time

Author, year	Time indicators
Capra et al. 2019 [18]	Reduction of OT set-up time to 7 months:
	hip = − 3 min, p = .06; knee = − 6 min, p = .01
Crosby et al. 2020 [19]	Reduced set-up time: 26–37% (p .05)
	Reduced set-up time: 58–66% (p .05)
Chin et al. 2014 [22]	Sterilisation time: 17.7''/instrument (range 7.6''–31.6'')
Dyas et al. 2018 [16]	Average set-up time in SO: 3 min (− 63%);
Farrokhi et al. 2013 [25]	Set-up time:
	MIS: − 4.9', 95% CI 2.1–7.7, p = .0015
	Average duration of procedure:
	MIS: − 7.2', 95% CI 3.8–10.7, p < .001
	Open spine: − 7.0', 95% CI 2.6–11.4, p = .0019
	Difference in duration between MIS and open spinal: p = .679
	DBS:
	electrode insertion: − 45', 95% CI − 9.2–99.4 p = .10
	Insertion of generator: − 11.5′ 95% CI 2.2–20.9 p = .018
	Generator revision: − 6.6′ 95% CI − 0.5–13.7 p = .066
Fu et al. 2021 [26]	OT set-up time: ò2l5 is/ -0.4 min (p = .03)
	Reprocessing time: − 1.4 is/− 0.2 min (p = .06)
Knowles et al. 2021 [30]	OT set-up time: − 2′42'' (vascular, p < .0001), − 3′57'' (aortic, p < .0001)
Koyle et al. 2015 [31]	Inventory cycle time: 5 min (vs. U,11 min; S,8 min)
Lonner et al. 2021 [32]	Reduced set-up time: 5.5’
	Reduced sterilisation time: − 40’/− 75’
Malone et al. 2018 [33]	Set-up time: − 43%
Marchand et al. 2020 [34]	Turnover time: -57 min
	Sterilisation time: − 40 min/set
Schwartz et al. 2021 [36]	Set-up time: − 22%
	Cleaning time: − 25%
Stockert and Langerman, 2014 [14]	Nurse time outside OT: 5.9–15.5%
	Average time surgeon idle: 9% of the intervention
Toor et al. 2022 [13]	Time saved on reprocessing: 1 333 h/year
Van Meter et al. 2016 [39]	Average OT time: 02:37 per individual case
Wannemuehler et al. 2015 [40]	Set-up time: − 44%
Wood et al. 2021 [41]	OT set-up time: − 2′12" to − 2′21" (p < .001)

**Table 6:** Cost-effectiveness of rationalising surgical sets

Author, year	Cost Indicators
Byrnes et al. 2017 [18]	Estimated savings in processing costs: $9.69/set
	Estimated total savings: 5.7%
Cichos et al. 2017 [15]	Estimated savings:
	thoracotomy, $96;
	robotic procedure, $84;
	mediastinoscopy, $77; VATS, $55
	Estimated total savings: $69 412/year
Cichos et al. 2019 [21]	Estimated average annual cost savings per set: $24 634
Dobson et al. 2015 [23]	Estimated savings: + 1.9% over the minimum theoretical cost
Dyas et al. 2018 [16]	Cost savings per intervention: $31.62 ($18.36-$49.98, reprocessing) + $100-$310 (reduced turnover time);
	Estimated annual savings: $28 458 (reprocessing) + $90 000-$2 790 000 (turnover time reduction)
Farrelly et al. 2017 [24]	Cost reduction:
	From labour: $3 523.28($-27.88; $2 014.26)
	(for 6 extensively used sets)
	by cost avoidance: $106 386 per turnover 5 years
	estimated savings of indirect costs: $34 693/year
Farrokhi et al. 2013 [25]	Estimated savings: -$2 800 000/year
Fu et al. 2021 [26]	Estimated savings: $9 010/year
Grodsky et al. 2020 [27]	estimated total annual savings per sterilisation: $9 588
John-Baptiste et al. 2016 [29]	$29 088/year
Knowles et al. 2021 [30]	Estimated annual savings: $97 781 (purchases), $97 444 (processing)
	Estimated savings of labour time: 316.2 h/year
Lonner et al. 2021 [32]	Estimated annual savings: $281 298.05
Malone et al. 2018 [33]	Reprocessing costs: -48% (-$23.97)
Marchand et al. 2020 [34]	Annual savings on OT and sterilisation centre processes: $249 245
Nast et al. 2019 [35]	estimated savings per set: $11.28–70.18
	estimated annual savings: $1 525.92–$11 531
Schwartz et al. 2021 [36]	Lower direct and indirect costs in post compared with that in pre: $93-$539 (p < .0001)
Shim et al. 2021 [37]	Estimated savings from replacement and processing costs: $6 597
Stockert and Langerman 2014 [14]	Estimated processing costs: $0.28–0.5/instrument
Thiel et al. 2019 [17]	Direct cost per case: -$125.44
Toor et al. 2022 [13]	Savings on depreciation costs: $61 395 (considering 8–12 years of use, depending on recommendations)
	Labour cost savings: $39 995/year
Tseng et al. 2020 [38]	Cost per case: -$123.24 (14.4%, p < .001)
	Total annual savings: $29 151
	Average cost difference between different surgeons: -61%
Van Meter et al. 2016 [39]	Total cost per instrument processing: annual $3.19 per instrument/year
	Estimation of excess cost from processing of unused instruments: $290 928/year
Wood et al. 2021 [41]	Estimated annual savings on instrument processing: $69 441

Therefore, the studies reported primary outcomes having an impact on the organisational framework.

18 studies indicated a reduction in the number of instruments from the original set to the optimised set. As five of these studies involved the optimisation of more than one set, they either reported a separate outcome for each set (n = 3) or an average value across sets (n = 2). Of the total number of items analysed, the percentage reduction in the number of instruments per individual set ranged from 19 to 89%.

In 10 studies, the observation phase in OT described the utilisation rate of the individual sets observed. Utilisation of instruments before optimisation ranged from 12.5% to 66%.

One study reported a comparison between the utilisation rate before and after the optimisation of two sets, showing an increase in use of between 27 and 30% [13].

One study analysed the difference between the perceived use of surgical devices and instruments by the surgeons involved and their actual use determined by observation, which were respectively 37% and 55% [12].

Seven studies measured the frequency of reopening the original set in a predetermined period after optimisation. Considering that a total of eight sets were optimised, in five instances no reopening events were recorded, in the other cases the frequency of reopening was 0.9%, 6% and 10% respectively.

One study measured a reduction in the frequency of procedure cancellations following the introduction of an optimised set: incidents dropped from 3.9% to 0.2% [16]. The same study submitted a questionnaire to gather indications of staff satisfaction with the instrumentation before and after optimisation, with an increase from 1.7% to 80% [13].

Eleven studies measured the time taken to set up the operating theatre, which decreased to 2 from 5 min following the introduction of optimised sets. Other studies observed that optimisation of the set had an impact on the overall duration of the procedure, with a 5 to 6-min reduction. A reduction in the time spent cleaning the operating theatre (-25%, n = 1), and time spent by the nurse on duty outside the theatre for reasons related to retrieval of surgical instruments (-15.5%, n = 1), were also observed. One study observed downtime of 9% of the entire procedure for reasons related to locating surgical instruments [14].

Concerning costs, many studies have estimated projected cost savings in the procurement, sterilisation and processing of instruments. The average annual savings were estimated at $1 525.00 to $2 800 000.00 (n = 12), depending on the activity flow of the various hospitals involved.

Differences in estimated savings were observed depending on the differing procedures: from $55.00 to $310.00 per single procedure [15–17].

## Discussion

The results of the systematic review brought to light observational studies that can be divided into two categories: evidence of purely clinical significance and evidence of mainly organisational, managerial, and financial significance. These two systems are largely interconnected, and reciprocally influence each other.

The decision to use disposable devices and instruments has been accompanied by a lower incidence in surgical site infections and surgical revisions for remediation. A concomitant reduction in post-operative functional recovery time has also been observed [11].

The rationalisation of traditional surgical sets has been observed in conjunction with outcomes of organisational significance, some of which could have an indirect clinical impact. As already highlighted by previous reviews, intra-operative time and time spent by nurses outside the room, as well as the reduction of setup time[7, 8] could lead to a further reduction in the time window of infection risk. Similarly, air movement in the operating room, potentially risky for contamination, could be reduced. However, studies reporting clinical outcomes as a direct consequence of organizational factors have not been found.

The effectiveness of the rationalisation of surgical sets seems to depend mainly on two conditions: the type of surgery, including the different access modes possible; and the organisational model followed. The main models observed in the interventions were Lean management and Kotter's change model. Responsibility for a training and awareness-raising process, with the active involvement of all stakeholders, appears to play a decisive role in practitioners’ participation in rationalisation practices and the long-term maintenance of the results obtained.

### Limits

The observational nature of all the studies identified means that the evaluations resulting from this review can be generalised taking into account the specific organisational context. This implies the need to promote future research on the topic by way of a context-specific analysis. In this context, research should consider the elements mentioned by Dos Santos and colleagues [6]: the promotion of increased consensus in multidisciplinary teams, participation in surgical set rationalisation projects and consolidation of the progress achieved; technologies for instrument tracking; a cross-sectional analysis of surgical sets aimed at instrument reduction; relocation of instruments excluded from sets; how to measure the 'non-material' and 'non-tangible' benefits of rationalisation and set safety after the process; and objectives following the establishment of a surgical set rationalisation cycle. Furthermore, clinical practice could benefit from primary or secondary research design that can exemplify outputs as a result of modelling that includes an analysis of clinical or organisational improvement in relation to specific organisational, cultural and functional frameworks. It should be noted that most of the quality improvement interventions analysed did not investigate the association with direct clinical outcomes on patients such as ISS incidence, mortality and morbidity, or the occurrence of other complications, due to the nature of the project, which is also exempt from ethical approval,due to the nature of the project which was also not accompanied by ethical approval. It is considered important that organisational process indicators go hand in hand in the future with an analysis of the direct effects in the clinical field, in order to further clarify the value of the processes considered.

Most of the studies were carried out in single centres. This implies, especially as regards rationalisation of surgical sets, that the results are extremely sensitive to the organisation of individual healthcare facilities and their catchment areas.

Cost estimates presented in studies have been developed retrospectively and in some cases according to direct case-by-case analysis. These projections may be sensitive to variables not examined, including the type and overall availability of instruments in the market, and may not be generalised regardless of the country in which the study was carried out.

## Data Availability

The datasets generated and analysed during the current study are not publicly available due, but are available from the corresponding author on reasonable request.

## MEDLINE

- 1. "Procedure"[Title/Abstract] OR "procedural"[Title/Abstract] OR "surgery"[Title/Abstract] OR "surgical"[Title/Abstract]
-   N = 2,559,850
- 2. "Tray"[Title/Abstract] OR "pack"[Title/Abstract]
-   N = 19.097
- 3. 1 and 2
-   N = 2.109
- 4. Limited to December 28th, 2021, English language, Humans
-   N = 1490

## EMBASE

- 1. "Procedure:ti,ab,kw OR procedural:ti,ab,kw OR surgery:ti,ab,kw N = 3482808
- 2. tray:ti,ab,kw OR pack:ti,ab,kw
- N = 30719
- 3. 1 AND 2
- N = 4364
- 4. Limited to Articles, English language, excluding MEDLINE records
- N = 273

## CINAHL

- 1. AB (Procedure or procedural or surgical) OR TI (Procedure or procedural or surgical)
- N=563200
- 2. AB (Tray or pack) OR TI (Tray or pack)
- N=7573
- 3. 1 and 2
- N=866
- 4. 3 limited to December 28th, 2021, English language, excluded MEDLINE records
- N=308

Permalink https://search.ebscohost.com/login.aspx?direct=true&AuthType=ip,sso&db=rzh&bquery=(+AB+(+Tray+or+pack+)+OR+TI+(+Tray+or+pack+)+)+AND+(+AB+(+Procedure+or+procedural+or+surgery+or+surgical+)+OR+TI+(+Procedure+or+procedural+or+surgery+or+surgical+)+)&cli0=DT1&clv0=000001202112&cli1=LA1&clv1=Y&cli2=MX1&clv2=Y&cli3=LA99&clv3=eng&type=1&searchMode=Standard&site=ehost-live&scope=site

## WEB OF SCIENCE

- 1. #1 AND #2
- 15,492
- Add to query
- 2
- TI = (tray or pack) OR AB = (tray or pack) OR KP = (tray or pack)
- 253,471
- Add to query
- 1
- TI = (Procedure or procedural or surgery or surgical) OR AB = (Procedure or procedural or surgery or surgical) OR KP = (Procedure or procedural or surgery or surgical)
- 3,261,025
- #1 AND #2 and Surgery (Web of Science Categories)
- 9:52 AM
- Web of Science Core Collection
- Show editions
- 2.607

**COCHRANE LIBRARY**

1. #1 (Procedure or procedural or surgical):ti,ab,kw N = 428,126
2. #2 (tray or pack):ti,ab,kw N = 4240
3. #3 #1 and #2 n = 1187

Last search: 12/22/2021 10:18

### Appendix 2. Bibliography of selected studies on full text

- Agarwal, R., N, Y. Sannappavar, Appukuttan, A., Ashok, A., &Rajanbabu, A. (2019). A prospective study evaluating the impact of implementing 'bundled interventions' in reducing surgical site infections among patients undergoing surgery for gynaecological Malignancies. *Eur J ObstetGynecolReprod Biol, 243*, 21-25. https://doi.org/10.1016/j.ejogrb.2019.10.007
- Akridge, J. (2004). Kits and trays: expanding on a value-driven product. *Healthcare Purchasing News, 28*(4), 16-20.
- Alfred, M., Catchpole, K., Huffer, E., Fredendall, L., & Taaffe, K. M. (2021). Work systems analysis of sterile processing: assembly. *BMJ Qual Saf, 30*(4), 271-282. https://doi.org/10.1136/bmjqs-2019-010740
- Arslan, F., &Yildiz, C. A. (2018). A Practical Suggestion for Prepare Dorsal Onlay Graft. *J Craniofac Surg, 29*(4), e344-e345. https://doi.org/10.1097/scs.0000000000004273
- Baskett, T. F. (2004). Surgical management of severe obstetric hemorrhage: experience with an obstetric hemorrhage equipment tray. *J ObstetGynaecol Can, 26*(9), 805-808. https://doi.org/10.1016/s1701-2163(16)30152-9
- Bhumisirikul, W., Bhumisirikul, P., &Pongchairerks, P. (2003). Long-term storage of small surgical instruments in autoclaved packages. *Asian J Surg, 26*(4), 202-204. https://doi.org/10.1016/s1015-9584(09)60303-1
- Blanc, E., Bombail, M., Magnin, S., Paysant-Vallas, M., Falquet, B., Rochefort, F., &Corvaisier, S. (2017). Quality of sterilization process evaluated with two new indicators: Accuracy of the information transmitted and impact on the process of surgical tray composition. *PharmacienHospitalier et Clinicien, 52*(1), 64-70. https://doi.org/10.1016/j.phclin.2016.07.008
- Bussières, M., L'Espèrance, S., Coulombe, M., &Rhainds, M. (2017). EVALUATION OF THE SURGICAL TRAY OPENING PROCEDURE IN OPERATING SUITES: SYSTEMATIC REVIEW AND RECOMMENDATIONS. *Ornac j, 35*(1), 57-66.
- Byrnes, Jenifer N., Schmitt, Jennifer J., Tommaso, Christopher P., &Occhino, John A. (2017). Cost reduction techniques in the operating suite: Surgical tray optimization: 72. *American Journal of Obstetrics and Gynecology, 216*, S616.
- Capra, R., Bini, S. A., Bowden, D. E., Etter, K., Callahan, M., Smith, R. T., & Vail, T. P. (2019). Implementing a perioperative efficiency initiative for orthopedic surgery instrumentation at an academic center: A comparative before-and-after study. *Medicine (Baltimore), 98*(7), e14338. https://doi.org/10.1097/md.0000000000014338
- Chin, Christopher J., Sowerby, Leigh J., John-Baptiste, Ava, & Rotenberg, Brian W. (2014). Reducing otolaryngology surgical inefficiency via assessment of tray redundancy. *Journal of Otolaryngology -- Head & Neck Surgery, 43*(1), 46-49. https://doi.org/10.1186/s40463-014-0046-2
- Cichos, K. H., Hyde, Z. B., Mabry, S. E., Ghanem, E. S., Brabston, E. W., Hayes, L. W., . . . Ponce, B. A. (2019). Optimization of Orthopedic Surgical Instrument Trays: Lean Principles to Reduce Fixed Operating Room Expenses. *J Arthroplasty, 34*(12), 2834-2840. https://doi.org/10.1016/j.arth.2019.07.040
- Cichos, K. H., Linsky, P. L., Wei, B., Minnich, D. J., &Cerfolio, R. J. (2017). Cost Savings of Standardization of Thoracic Surgical Instruments: The Process of Lean. *Ann Thorac Surg, 104*(6), 1889-1895. https://doi.org/10.1016/j.athoracsur.2017.06.064
- Costa, D. M., Lopes, L. K. O., Vickery, K., Watanabe, E., Vasconcelos, Lsnol, de Paula, M. C., . . . Tipple, A. F. V. (2018). Reprocessing safety issues associated with complex-design orthopaedic loaned surgical instruments and implants. *Injury, 49*(11), 2005-2012. https://doi.org/10.1016/j.injury.2018.09.006
- Crosby, L., Lortie, E., Rotenberg, B., & Sowerby, L. (2020). Surgical Instrument Optimization to Reduce Instrument Processing and Operating Room Setup Time. *Otolaryngol Head Neck Surg, 162*(2), 215-219. https://doi.org/10.1177/0194599819885635
- Dekonenko, C., Oyetunji, T. A., &Rentea, R. M. (2020). Surgical tray reduction for cost saving in pediatric surgical cases: A qualitative systematic review. *J Pediatr Surg, 55*(11), 2435-2441. https://doi.org/10.1016/j.jpedsurg.2020.05.010
- Dieryck, K., Mertens, W., &Zeyen, T. (1998). Costsaving in the operating room: the Procedure Pack. *Bull Soc BelgeOphtalmol, 270*, 47-49.
- Dilworth, J. P., & White, R. J. (1992). Postoperative chest infection after upper abdominal surgery: an important problem for smokers. *Respir Med, 86*(3), 205-210. https://doi.org/10.1016/s0954-6111(06)80056-9
- Dobson, G., Seidmann, A., Tilson, V., &Froix, A. (2015). Configuring surgical instrument trays to reduce costs. *IIE Transactions on Healthcare Systems Engineering, 5*(4), 225-237. https://doi.org/10.1080/19488300.2015.1094759
- Dos Santos, B. M., Fogliatto, F. S., Zani, C. M., & Peres, F. A. P. (2021). Approaches to the rationalization of surgical instrument trays: scoping review and research agenda. *BMC Health Serv Res, 21*(1), 163. https://doi.org/10.1186/s12913-021-06142-8
- Dyas, A. R., Lovell, K. M., Balentine, C. J., Wang, T. N., Porterfield, J. R., Jr., Chen, H., & Lindeman, B. M. (2018). Reducing cost and improving operating room efficiency: examination of surgical instrument processing. *J Surg Res, 229*, 15-19. https://doi.org/10.1016/j.jss.2018.03.038
- Edlich, R. F., Jones, K. C., Jr., Buchanan, L., Morgan, R. G., McGregor, W., &Himel, H. N. (1992). A disposable emergency wound treatment kit. *J Emerg Med, 10*(4), 463-467. https://doi.org/10.1016/0736-4679(92)90276-y
- Egan, P., Pierce, A., Flynn, A., Teeling, S. P., Ward, M., & McNamara, M. (2021). Releasing operating room nursing time to care through the reduction of surgical case preparation time: A lean six sigma pilot study. *International Journal of Environmental Research and Public Health, 18*(22). https://doi.org/10.3390/ijerph182212098
- Eggleston, M. K., Jr., Wax, J. R., Philput, C., Eggleston, M. H., & Weiss, M. I. (1997). Use of surgical pass trays to reduce intraoperative glove perforations. *J Matern Fetal Med, 6*(4), 245-247.
- Fadaak, R., Davies, J. M., Blaak, M. J., Conly, J., Haslock, J., Kenny, A., . . . Leslie, M. (2021). Rapid conversion of an in-patient hospital unit to accommodate COVID-19: An interdisciplinary human factors, ethnography, and infection prevention and control approach. *PLoS One, 16*(1), e0245212. https://doi.org/10.1371/journal.pone.0245212
- Farrelly, J. S., Clemons, C., Witkins, S., Hall, W., Christison-Lagay, E. R., Ozgediz, D. E., . . . Caty, M. G. (2017). Surgical tray optimization as a simple means to decrease perioperative costs. *Journal of Surgical Research, 220*, 320-326. https://doi.org/10.1016/j.jss.2017.06.029
- Farrokhi, F. R., Gunther, M., Williams, B., & Blackmore, C. C. (2015). Application of Lean Methodology for Improved Quality and Efficiency in Operating Room Instrument Availability. *J Healthc Qual, 37*(5), 277-286. https://doi.org/10.1111/jhq.12053
- Fogliatto, F. S., Anzanello, M. J., Tortorella, G. L., Schneider, D. S. S., Pereira, C. G. R., & Schaan, B. D. (2018). A Six Sigma Approach to Analyze Time-to-Assembly Variance of Surgical Trays in a Sterile Services Department. *J Healthc Qual, 40*(3), e46-e53. https://doi.org/10.1097/jhq.0000000000000078
- Glaser, B., Dänzer, S., &Neumuth, T. (2015). Intra-operative surgical instrument usage detection on a multi-sensor table. *Int J Comput Assist Radiol Surg, 10*(3), 351-362. https://doi.org/10.1007/s11548-014-1066-0
- Grodsky, J. D., Theophanous, C. N., Schechet, S. A., Veldman, P. B., &Hariprasad, S. M. (2020). Reducing instruments in a vitrectomy surgical tray: Cost savings and results from a major academic hospital. *International Journal of Retina and Vitreous, 6*(1). https://doi.org/10.1186/s40942-020-00215-2
- Halbert, R. J., Simon, R. R., &Leshuk, L. (1988). Logistics of medical care in rural Afghanistan. *Ann Emerg Med, 17*(8), 770-774. https://doi.org/10.1016/s0196-0644(88)80549-3
- Harvey, L., Slocum, P., Heft, J., Van Meter, M., Lovett, B., & Adam, R. (2017). Gynecologic Surgery Instrument Trays: Leveraging Surgeon Knowledge to Improve Supply Chain Efficiency. *Journal of Gynecologic Surgery, 33*(5), 180-183. https://doi.org/10.1089/gyn.2017.0039
- Haya, N., Feiner, B., Baessler, K., Christmann-Schmid, C., & Maher, C. (2018). Perioperative interventions in pelvic organ prolapse surgery. *Cochrane Database Syst Rev, 8*(8), Cd013105. https://doi.org/10.1002/14651858.cd013105
- Holdsworth, Jill. (2021). Targeting Zero—preventing Surgical Site Infections by Reducing Immediate Use Steam Sterilization (IUSS) of Surgical Instruments...Association for Professionals in Infection Control and Epidemiology, Annual Conference (Virtual), 28-30 June, 2021. *American Journal of Infection Control, 49*(6), S5-S5. https://doi.org/10.1016/j.ajic.2021.04.019
- Huang, Y., Chen, Y., Pan, W., Hu, J., & Yi, L. (2021). Factors affecting implementation and pass rates of surgical instrument moistening. *BMC Infect Dis, 21*(1), 752. https://doi.org/10.1186/s12879-021-06471-3
- Igesund, Unni, Overvåg, Grete, Rasmussen, Guri, &Rekvig, Ole Petter. (2019). Mapping of procedures for set-up of instruments in the sterile field for surgery. *Norwegian Journal of Clinical Nursing / SykepleienForskning*, 1-24. https://doi.org/10.4220/Sykepleienf.2019.78413
- John-Baptiste, A., Sowerby, L. J., Chin, C. J., Martin, J., & Rotenberg, B. W. (2016). Comparing surgical trays with redundant instruments with trays with reduced instruments: a cost analysis. *CMAJ Open, 4*(3), E404-e408. https://doi.org/10.9778/cmajo.20150092
- Kaygusuz, I., Kizirgil, A., Karlidağ, T., Yalçin, S., Keles, E., Yakupoğullari, Y., &Alpay, C. (2003). Bacteriemia in septoplasty and septorhinoplasty surgery. *Rhinology, 41*(2), 76-79.
- Khurana, S., Saini, S. S., Sundaram, V., Dutta, S., & Kumar, P. (2018). Reducing Healthcare-associated Infections in Neonates by Standardizing and Improving Compliance to Aseptic Non-touch Techniques: A Quality Improvement Approach. *Indian Pediatr, 55*(9), 748-752.
- Kirmse, G., & Graf, M. (2021). Comparison of the cleaning and drying performance of different instrument tray designs and accessories. *Zentralsterilisation - Central Service, 29*(1), 42-47.
- Knowles, M., Gay, S. S., Konchan, S. K., Mendes, R., Rath, S., Deshpande, V., . . . Wood, B. C. (2021). Data analysis of vascular surgery instrument trays yielded large cost and efficiency savings. *J Vasc Surg, 73*(6), 2144-2153. https://doi.org/10.1016/j.jvs.2020.09.043
- Kong, K. C., Sheppard, M., &Serne, G. (1994). Dispensing surgical gloves onto the open surgical gown pack does not increase the bacterial contamination rate. *J Hosp Infect, 26*(4), 293-296. https://doi.org/10.1016/0195-6701(94)90020-5
- Koyle, M. A., AlQarni, N., Odeh, R., Butt, H., Alkahtani, M. M., Konstant, L., . . . Baker, G. R. (2018). Reduction and standardization of surgical instruments in pediatric inguinal hernia repair. *J PediatrUrol, 14*(1), 20-24. https://doi.org/10.1016/j.jpurol.2017.08.002
- Kusuda, K., Yamashita, K., Ohnishi, A., Tanaka, K., Komino, M., Honda, H., . . . Ohta, Y. (2016). Management of surgical instruments with radio frequency identification tags. *Int J Health Care Qual Assur, 29*(2), 236-247. https://doi.org/10.1108/ijhcqa-03-2015-0034
- Kwaan, M. R., Weight, C. J., Carda, S. J., Mills-Hokanson, A., Wood, E., Rivard-Hunt, C., &Argenta, P. A. (2016). Abdominal closure protocol in colorectal, gynecologic oncology, and urology procedures: a randomized quality improvement trial. *Am J Surg, 211*(6), 1077-1083. https://doi.org/10.1016/j.amjsurg.2015.10.032
- Lin, D. M., Carson, K. A., Lubomski, L. H., Wick, E. C., & Pham, J. C. (2018). Statewide Collaborative to Reduce Surgical Site Infections: Results of the Hawaii Surgical Unit-Based Safety Program. *J Am Coll Surg, 227*(2), 189-197.e181. https://doi.org/10.1016/j.jamcollsurg.2018.04.031
- Litrico, Stéphane, Recanati, Geoffrey, Gennari, Antoine, Maillot, Cédric, Saffarini, Mo, &Huec, Jean-Charles. (2016). Single-use instrumentation in posterior lumbar fusion could decrease incidence of surgical site infection: a prospective bi-centric study. *European Journal of Orthopaedic Surgery & Traumatology, 26*(1), 21-26. https://doi.org/10.1007/s00590-015-1692-4
- Littell, J. J. (1951). New technique in the use of a surgical tray. *AMA Arch Otolaryngol, 54*(2), 195-197.
- Lonner, J. H., Goh, G. S., Sommer, K., Niggeman, G., Levicoff, E. A., Vernace, J. V., & Good, R. P. (2021). Minimizing Surgical Instrument Burden Increases Operating Room Efficiency and Reduces Perioperative Costs in Total Joint Arthroplasty. *J Arthroplasty, 36*(6), 1857-1863. https://doi.org/10.1016/j.arth.2021.01.041
- Malone, E., Baldwin, J., Richman, J., Lancaster, R., Krontiras, H., & Parker, C. (2019). The Impact of Breast Lumpectomy Tray Utilization on Cost Savings. *J Surg Res, 233*, 32-35. https://doi.org/10.1016/j.jss.2018.06.063
- Marchand, K. B., Taylor, K. B., Salem, H. S., Mont, M. A., & Marchand, R. C. (2020). Surgical Tray Optimization and Efficiency: The Impact of a Novel Sealed Sterile Container and Instrument Tray Technology. *Surg Technol Int, 37*, 349-355.
- Martinez, C., Omesiete, P., Pandit, V., Thompson, E., Nocera, M., Riall, T., . . . Nfonsam, V. (2020). A Protocol-Driven Reduction in Surgical Site Infections After Colon Surgery. *J Surg Res, 246*, 100-105. https://doi.org/10.1016/j.jss.2019.08.018
- McDermott, A. M., O'Cathain, E., Carey, B. W., O'Sullivan, P., & Sheahan, P. (2016). Sphenopalatine Artery Ligation for Epistaxis: Factors Influencing Outcome and Impact of Timing of Surgery. *Otolaryngol Head Neck Surg, 154*(3), 547-552. https://doi.org/10.1177/0194599815620134
- Meals, C. G., & Meals, R. A. (2007). A history of surgery in the instrument tray: eponymous tools used in hand surgery. *J Hand SurgAm, 32*(7), 942-953. https://doi.org/10.1016/j.jhsa.2007.05.007
- Moccia, G., Motta, O., Pironti, C., Proto, A., Capunzo, M., & De Caro, F. (2020). An alternative approach for the decontamination of hospital settings. *J Infect Public Health, 13*(12), 2038-2044. https://doi.org/10.1016/j.jiph.2020.09.020
- Nadeau, Kara. (2018). The quest for consistency: Product and process standardization drives effective kit/procedure tray planning. *Healthcare Purchasing News, 42*(11), 18-20.
- Nast, K., & Swords, K. A. (2019). Decreasing operating room costs via reduction of surgical instruments. *J PediatrUrol, 15*(2), 153.e151-153.e156. https://doi.org/10.1016/j.jpurol.2019.01.013
- Navi, Fina, Motamedy, Mohammad HoseinKalantar, Faiaz, Fariba, Valayi, Naser, Lasemi, Eshagh, Hovakhti, Afagh, &Alavi, Amin. (2012). Action research for controlling bacterial contamination of surgery Sets sterilized by CSR war autoclave. *Journal of Research in Dental Sciences, 9*(2), 1p-1p.
- Nguyen, J. M. V., Sadeghi, M., Gien, L. T., Covens, A., Kupets, R., Nathens, A. B., & Vicus, D. (2019). Impact of a preventive bundle to reduce surgical site infections in gynecologic oncology. *Gynecol Oncol, 152*(3), 480-485. https://doi.org/10.1016/j.ygyno.2018.09.008
- O'Donnell, P. (2002). Viewpoint. Taking control of custom procedure packs. *SSM, 8*(3), 16-18.
- Osborn, L. K., & Hertz, E. (1999). Revising surgical instrument trays. *Surgical Services Management, 5*(6), 35-38.
- Panahi, P., Stroh, M., Casper, D. S., Parvizi, J., & Austin, M. S. (2012). Operating room traffic is a major concern during total joint arthroplasty. *Clin OrthopRelat Res, 470*(10), 2690-2694. https://doi.org/10.1007/s11999-012-2252-4
- Parker, P. J. (2006). (ii) Initial medical and surgical management. *Current Orthopaedics, 20*(5), 333-395.
- Passey, A. (2002). Custom procedure trays in Europe: the strategic challenges. *News Review (09637974)*(138), 10-10.
- Rao, M. K., Reilley, T. E., Schuller, D. E., & Young, D. C. (1992). Analysis of risk factors for postoperative pulmonary complications in head and neck surgery. *Laryngoscope, 102*(1), 45-47. https://doi.org/10.1288/00005537-199201000-00008
- Reams, C. A. (2013). What's on your surgical tray? *Journal of the Dermatology Nurses' Association, 5*(6), 346-347. https://doi.org/10.1097/JDN.0000000000000003
- Schiavone, M. B., Moukarzel, L., Leong, K., Zhou, Q. C., Afonso, A. M., Iasonos, A., . . . Zivanovic, O. (2017). Surgical site infection reduction bundle in patients with gynecologic cancer undergoing colon surgery. *Gynecol Oncol, 147*(1), 115-119. https://doi.org/10.1016/j.ygyno.2017.07.010
- Schwartz, J. L., Kirkpatrick, L., Hillebrecht, K. E., Lee, J. S., Steiman, J. G., Soran, A., Diego, E. J. (2021). Cutting Instruments to Cut Costs: A Simple Initiative with Breast Surgical Operating Room Trays that Resulted in Substantial Savings. *Ann Surg Oncol, 28*(10), 5553-5557. https://doi.org/10.1245/s10434-021-10496-y
- Schömig, F., Perka, C., Pumberger, M., &Ascherl, R. (2020). Implant contamination as a cause of surgical site infection in spinal surgery: are single-use implants a reasonable solution? - a systematic review. *BMC MusculoskeletDisord, 21*(1), 634. https://doi.org/10.1186/s12891-020-03653-z
- Sebben, J. E. (1988). Sterile technique and the prevention of wound infection in office surgery--Part I. *J Dermatol Surg Oncol, 14*(12), 1364-1371. https://doi.org/10.1111/j.1524-4725.1988.tb01127.x
- Sheth, S. S. (2003). Pneumo-surgical pack-a preliminary report. *Int J GynaecolObstet, 81*(2), 213-215. https://doi.org/10.1016/s0020-7292(03)00078-x
- Shim, S. S., Danford, N. C., Wright, M. L., Abernathie, L. A. V., Kim, J. S., Kadiyala, R. K., &Vosseller, J. T. (2021). Economic Impact of Unused Surgical Instruments in an Orthopaedic Surgery Department at an Academic Medical Center: A Prospective Cross-sectional Study. *J Surg Orthop Adv, 30*(3), 131-135.
- Siegel, G. W., Patel, N. N., Milshteyn, M. A., Buzas, D., Lombardo, D. J., &Morawa, L. G. (2015). Cost Analysis and Surgical Site Infection Rates in Total Knee Arthroplasty Comparing Traditional vs. Single-Use Instrumentation. *J Arthroplasty, 30*(12), 2271-2274. https://doi.org/10.1016/j.arth.2015.05.037
- Sterne JAC, Savović J, Page MJ, Elbers RG, Blencowe NS, Boutron I, Cates CJ, Cheng HY, Corbett MS, Eldridge SM, Emberson JR, Hernán MA, Hopewell S, Hróbjartsson A, Junqueira DR, Jüni P, Kirkham JJ, Lasserson T, Li T, McAleenan A, Reeves BC, Shepperd S, Shrier I, Stewart LA, Tilling K, White IR, Whiting PF, Higgins JPT. RoB 2: a revised tool for assessing risk of bias in randomised trials. BMJ. 2019 Aug 28;366:l4898. https://doi.org/10.1136/bmj.l4898. PMID: 31462531.
- Stockert, E. W., &Langerman, A. (2014). Assessing the magnitude and costs of intraoperative inefficiencies attributable to surgical instrument trays. *Journal of the American College of Surgeons, 219*(4), 646-655. https://doi.org/10.1016/j.jamcollsurg.2014.06.019
- Strulak, L., Gronki, F., Shariat, K., Schöni, D., & Alfieri, A. (2021). Eponyms of Cranial Neurosurgical Instruments: An International Collaboration to Optimize the Field of Neurosurgery. *World Neurosurg, 153*, 26-35. https://doi.org/10.1016/j.wneu.2021.06.073
- Strzelecki, L. R., & Nelson, J. H. (1989). Evaluation of closed container flash sterilization system. *OrthopNurs, 8*(1), 21-24. https://doi.org/10.1097/00006416-198901000-00006
- Thiel, C. L., Fiorin Carvalho, R., Hess, L., Tighe, J., Laurence, V., Bilec, M. M., &Baratz, M. (2019). Minimal Custom Pack Design and Wide-Awake Hand Surgery: Reducing Waste and Spending in the Orthopedic Operating Room. *Hand (N Y), 14*(2), 271-276. https://doi.org/10.1177/1558944717743595
- Torres, A., Inzunza, M., Jarry, C., Serrano, F., Varas, J., & Zavala, A. (2021). DEVELOPMENT AND VALIDATION OF A NEW LAPAROSCOPIC ENDOTRAINER FOR NEONATAL SURGERY AND REDUCED SPACES. *Arq Bras Cir Dig, 33*(4), e1559. https://doi.org/10.1590/0102-672020200004e1559
- Tseng, J., Sax, H. C., Gewertz, B. L., Margulies, D. R., & Alban, R. F. (2020). Surgical Supply Cost Awareness Is Associated With Lower Costs: A Single-Center Experience. *Am Surg, 86*(10), 1407-1410. https://doi.org/10.1177/0003134820964494
- Van Meter, M. M., & Adam, R. A. (2016). Costs associated with instrument sterilization in gynecologic surgery. *Am J ObstetGynecol, 215*(5), 652.e651-652.e655. https://doi.org/10.1016/j.ajog.2016.06.019
- Wagner, J. A., Dexter, F., Greeley, D. G., & Schreiber, K. (2021). Operating room air delivery design to protect patient and surgical site results in particles released at surgical table having greater concentration along walls of the room than at the instrument tray. *Am J Infect Control, 49*(5), 593-596. https://doi.org/10.1016/j.ajic.2020.10.003
- Wannemuehler, T. J., Elghouche, A. N., Kokoska, M. S., Deig, C. R., & Matt, B. H. (2015). Impact of Lean on surgical instrument reduction: Less is more. *Laryngoscope, 125*(12), 2810-2815. https://doi.org/10.1002/lary.25407
- Watters, T. S., Mather, R. C., 3rd, Browne, J. A., Berend, K. R., Lombardi, A. V., Jr., &Bolognesi, M. P. (2011). Analysis of procedure-related costs and proposed benefits of using patient-specific approach in total knee arthroplasty. *J Surg Orthop Adv, 20*(2), 112-116.
- Weber, L. A. (1998). The surgical tray. *Dermatol Clin, 16*(1), 17-24. https://doi.org/10.1016/s0733-8635(05)70484-8
- Weiser, M. C., Shemesh, S., Chen, D. D., Bronson, M. J., &Moucha, C. S. (2018). The Effect of Door Opening on Positive Pressure and Airflow in Operating Rooms. *J Am AcadOrthop Surg, 26*(5), e105-e113. https://doi.org/10.5435/jaaos-d-16-00891
- Wells, Rick. (2017). Can using custom trays reduce instrument repairs? *Healthcare Purchasing News, 41*(6), 78-79.
- Wells, Rick. (2018). Hospital improves costs, efficiencies with custom trays: Part 2: Study completion delivers impressive results. *Healthcare Purchasing News, 42*(5), 60-61.
- Wood, B. C., Konchan, S., Gay, S., Rath, S., Deshpande, V., & Knowles, M. (2021). Data Analysis of Plastic Surgery Instrument Trays Yields Significant Cost Savings and Efficiency Gains. *Ann Plast Surg, 86*(6S Suppl 5), S635-s639. https://doi.org/10.1097/sap.0000000000002913

### Appendix 2.1: Excluded articles with cause

- Full text non disponibile (9): Littell 1951, Sebben 1988, Sheth 2003, Dieryck 1998, Stephanie 2010, Akridge 2004, Osborne 1999, Passey 2002, Wilkie 1986
- Outcome non descritti o non d’interesse: (24) Ahmadi, 2019; Egan 2021, Baskett 2004, Bhumisorikul 2004, Costa 2018, Fogliatto 2018, Glaser 2015, Halbert 1988, Huang 2021, Kimse 2021, Kusuda 2016, Strulak 2021; Strzelecki, 1989, Torres 2021, Bradley 2019, Alfred 2021, Dana Barlow 2015, Holdsworth 2021, Igesund 2019, Navi 2012, Parker 2006, Wells 2017, Wells 2018, Eggleston 1997
- Non focus su set chirurgici: (24) Agarwal 2019, Arslan 2018, Dilworth 1992, Edilich 1992, Fadaak 2021, Haya 2018, Johnson 2016, Kaygusuz 2003, Khurana 2018, Kong 1994; Kwaan 2016; Lin 2018, Martinez 2020; McDermott 2016; Meals 2007; Moccia 2020; Nguyen 2019; Panahi 2012; Rao 1992, Wagner 2021, Weiser 2018; Watters, 2011; Makram 2021, Olivere 2021
- – Study design not contemplated (es. editoriali, case reports, opinion papers) (6): Reams 2013, Weber 1998, Nadeau 2018, O’Donnell 2002, Stephanie 2010, Goldberg 2019
- Full text in altra lingua (1): Blanc 2017
